# Towards subject-level cerebral infarction classification of CT scans using convolutional networks

**DOI:** 10.1371/journal.pone.0235765

**Published:** 2020-07-15

**Authors:** Manuel Schultheiss, Peter B. Noël, Isabelle Riederer, Frank Thiele, Felix K. Kopp, Bernhard Renger, Ernst J. Rummeny, Franz Pfeiffer, Daniela Pfeiffer

**Affiliations:** 1 Department of Diagnostic and Interventional Radiology, School of Medicine & Klinikum rechts der Isar, Technical University of Munich, München, Germany; 2 Chair of Biomedical Physics, Department of Physics and Munich School of BioEngineering, Technical University of Munich, Garching, Germany; 3 Philips Research Laboratories, Aachen, Germany; 4 Institute for Advanced Study, Technical University of Munich, Garching, Germany; 5 Department of Diagnostic and Interventional Neuroradiology, School of Medicine & Klinikum rechts der Isar, Technical University of Munich, München, Germany; Politechnika Krakowska im Tadeusza Kosciuszki, POLAND

## Abstract

Automatic evaluation of 3D volumes is a topic of importance in order to speed up clinical decision making. We describe a method to classify computed tomography scans on volume level for the presence of non-acute cerebral infarction. This is not a trivial task, as the lesions are often similar to other areas in the brain regarding shape and intensity. A three stage architecture is used for classification: 1) A cranial cavity segmentation network is developed, trained and applied. 2) Region proposals are generated 3) Connected regions are classified using a multi-resolution, densely connected 3D convolutional network. Mean area under curve values for subject level classification are 0.95 for the unstratified test set, 0.88 for stratification by patient age and 0.93 for stratification by CT scanner model. We use a partly segmented dataset of 555 scans of which 186 scans are used in the unstratified test set. Furthermore we examine possible dataset bias for scanner model and patient age parameters. We show a successful application of the proposed three-stage model for full volume classification. In contrast to black-box approaches, the convolutional network’s decision can be further assessed by examination of intermediate segmentation results.

## Introduction

Stroke is one of the leading death causes world-wide [1]. It can be further differentiated between hemorrhagic stroke and ischemic stroke, whereby ischemic stroke occurs around 5.5 times more often and has a lower mortality rate, however, functional independence of patients is often inhibited which makes stroke rehabilitation necessary. Immediate diagnosis to start treatment is important and usually supported by computed tomography (CT) or magnetic resonance imaging (MRI). [2]

In our work, we focus on ischemic stroke areas that hipoattenuate in CT [3], in which neurons typically undergo cell necrosis or apoptosis [4, 5]. Diagnosis of medical images can be supported by computer aided diagnosis (CAD), which is a strategy to reduce the probability of missing abnormalities [6]. A considerable amount of literature about the application of CAD for automated stroke lesion segmentation has been published lately: Non deep-learning approaches make use of hand-crafted features such as histograms [7], local entropy or median intensities [8]. There are several publications on stroke lesion segmentation from MRI images which either utilize a U-Net [9] like architecture [10, 11] or classify voxels on patch level [12]. For thrombus detection in CT images, Lisowska et al. [13] also utilized a patch based approach with additional atlas coordinates. There are also challenges focusing on segmentation, however, these only use a subset of slices from the whole CT scan for evaluation [14] and do not provide healthy subjects in their datasets. For clinical decision making, classification on volume level is of more interest, as it does not require to manually select a region of interest. Recently, techniques for optical coherence tomography (OCT) or CT volume classification, which still make use of slice-wise or voxel-wise annotation, but output predictions on volume level, have been proposed [15–18]. Apart from that, convolutional neural networks (CNNs) which only make use of volume-wise, binary annotation have also been evaluated for hemorrhage detection [19]. However, these require a large number (N = 37074) of training samples. Recent work also highlights the importance of result explanation for deep learning systems applied in a clinical environment [16, 18].

Our contributions are as follows: 1) We present a fully automatic subject level classification method of patients with cerebral infarction, which does not require to select a region of interest manually and is evaluated on a test-set with healthy and diseased patients. 2) We compare our algorithm with existing approaches for 3D volume classification 3) We present a method for cranial cavity segmentation of CT scans that is, to the best of our knowledge, faster than existing methods and evaluate its use for stroke classification 4) We investigate if our method is prone to dataset bias.

For the initial development and testing of the algorithm, we focused on non-acute stroke lesions. Hereby, the ground-truth can be retrieved more easily than for acute stroke volumes. However, the clinical use case is limited to structured reporting. For the classification of acute stroke cases, an algorithm similar to the presented one could be used. Hence, this work lies the foundation for the detection of acute cases.

The paper is structured as follows: in the *Material and Methods* section the dataset, the used convolutional network architectures and the network training procedure is described. The *Results* section reports results for the classification network and intermediate results for segmentation networks. The *Discussion* section puts the results in context with current literature and highlights advantages and disadvantages of the proposed method.

## Materials and methods

**Fig 1:**
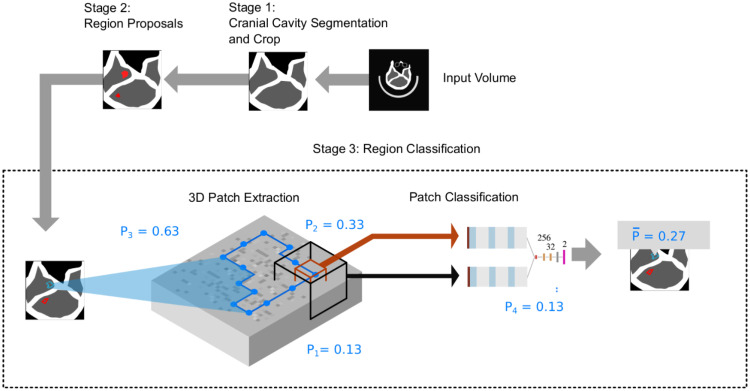
Illustration of inference steps for classification of test set volumes. First the cranial cavity segmentation is performed. To generate region proposals, the region containing the cranial cavity is given as input to the stage 2 network. The output is a segmentation of possible stroke lesions. Next, patches (orange and black boxes) are extracted from the original volume with positions corresponding to the border of the segmentations (blue). Hereafter, the patches are given as input to the stage 3 network, which yields a probability for each patch. Lesion probabilities are calculated by averaging the probabilities of patches on that lesion. Subject-level probability is later determined by calculating the maximum of all lesion probabilities within a scan.

The main objective of this work was to provide a reliable, automatic pipeline for cerebral infarction classification of non-contrast CT scans, which is also capable to provide visual explanations. We use a three stage approach, which systematically reduces the amount of voxels used for final classification: after preprocessing, a cranial cavity segmentation to extract brain tissue is performed in stage 1. In the second stage, a region proposals network is trained on slice level, which proposes regions where stroke is to occur most likely, but does not provide an individual probability for each region yet. Hereby a high sensitivity of the network is desirable, as false positives are eliminated in the third stage. In this stage, a network differentiating between patches extracted from the ground-truth radiologist annotations and patches extracted from region proposals of healthy volumes is trained. For inference, the three stages are run through consecutively (Fig 1).

### Dataset description

CT scans of 555 patients were retrospectively collected at our institution. Of these, 171 patients had a confirmed non-acute stroke diagnosis with hipoattenuating tissue visible in the CT scan. Data access was approved by the institutional ethics committee at Klinikum Rechts der Isar (Ethikvotum 87/18 S) and data was anonymized. The ethics committee has waived the need for informed consent. All research was performed in accordance with relevant guidelines and regulations. Reference standard for diseased patients was obtained based on the diagnosis of two radiologists, while reference standard for healthy patients was based on the diagnosis of one radiologist. Segmentation of the stroke lesions was performed by a radiologist using an in-house annotation software.

Slice thickness of the scans was 5mm for 435 scans. 109 scans were acquired with two different slice thicknesses (3mm and 6mm) within a single cranial scan. Further 11 scans were acquired with 6mm, 3mm, 4mm or 8mm slice thickness. From the patients, 284 patients were male and 271 patients were female. Mean patient age was 67.02±17.05 years. Scanner and age distribution is shown in Table 1.

**Table 1:** Dataset split.

	S, All	H, All	S, Test No Strat.	H, Test No Strat.	S, Test Age	H, Test Age	S, Test Scanner	H, Test Scanner
10-20	0	3	0	1	0	0	0	2
20-30	0	20	0	8	0	0	0	10
30-40	1	24	0	7	0	0	0	3
40-50	5	40	3	13	3	3	3	13
50-60	13	52	9	13	9	9	7	14
60-70	33	74	16	17	16	16	9	11
70-80	56	100	32	20	30	30	28	11
80-90	54	51	28	9	24	24	24	7
90-100	9	19	5	4	4	4	4	4
100-110	0	1	0	1	0	0	0	0
Brilliance 64	1	2	1	1	1	0	1	1
Brilliance iCT	3	0	3	0	3	0	0	0
iCT 256	33	71	13	14	12	17	10	10
Ingenuity CT	2	0	2	0	2	0	0	0
IQon—Spectral CT	22	26	16	16	14	9	16	16
Sensation 16	9	13	6	2	5	3	6	6
Sensation Cardiac 64	44	49	22	8	20	4	22	22
SOMATOM Definition AS	30	208	13	40	13	47	13	13
SOMATOM Definition AS+	22	15	13	12	12	6	7	7
Volume Zoom	5	0	4	0	4	0	0	0

Detailed dataset split. *S* indicate stroke patients and *H* indicate healthy patients. For stratified test sets, patient occurrences are balanced by CT scanner model and age.

**Fig 2:**
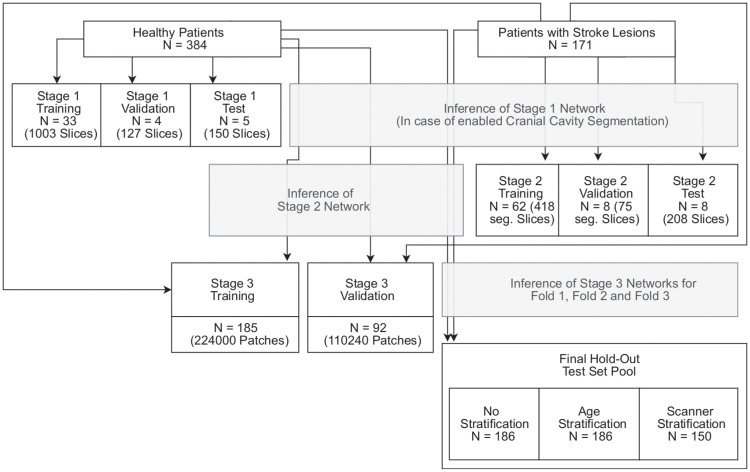
Overview of data-flow. Arrows passing a box indicate inference of a trained network. For stage 2 training and validation, only segmented slices containing a stroke lesion were used (abbreviated with seg.). Dependent on stratification, a different number of patients was used for the last stage hold-out test-set. None of the final hold-out test-set patients was used in a previous stage for training, validation or test.

For training of the cranial cavity segmentation network, the cranial cavity of 42 healthy patients was segmented. Overall data flow is illustrated in Fig 2. For each of the three stages, the training/validation/test data split is described in the respective section.

In case of primary or secondary non-stroke like pathologies or abnormalities, CT scans were excluded from the dataset.

### Data extraction and preprocessing

DICOM pixel data is read using the pydicom library [20] and slices are assembled to Numpy based 3D ndarray [21] volumes. Slice positions are determined by sorting slices by the z-coordinate of the *ImagePositionPatient* DICOM tag. Intensity values below 0 HU and above 80 HU are clipped to match the stroke window radiologists at our institution use for diagnosis. As network training input requires values to be in a range between 0 and 1, HU values are normalized using a min-max normlization scheme. In our case this results in a division by 80, which is the maximum HU value.

After cranial cavity segmentation, rotation of the head is performed on the x-y plane in order to align the mid-line axis, similar to [7]. Areas with a density below water (HU ≤0) surrounding the cranial cavity are cropped.

### Cranial cavity segmentation

Prior to the region proposal generation the cranial cavity, which contains the brain, needs to be segmented. Organ of interest segmentation was also utilized in previous literature [22–24]. Hereby, for CT scans, the number of voxels can be limited by removing irrelevant objects like the patient table from the scan. Hence, the memory usage and training time of the consecutive CNNs can be reduced.

**Fig 3:**
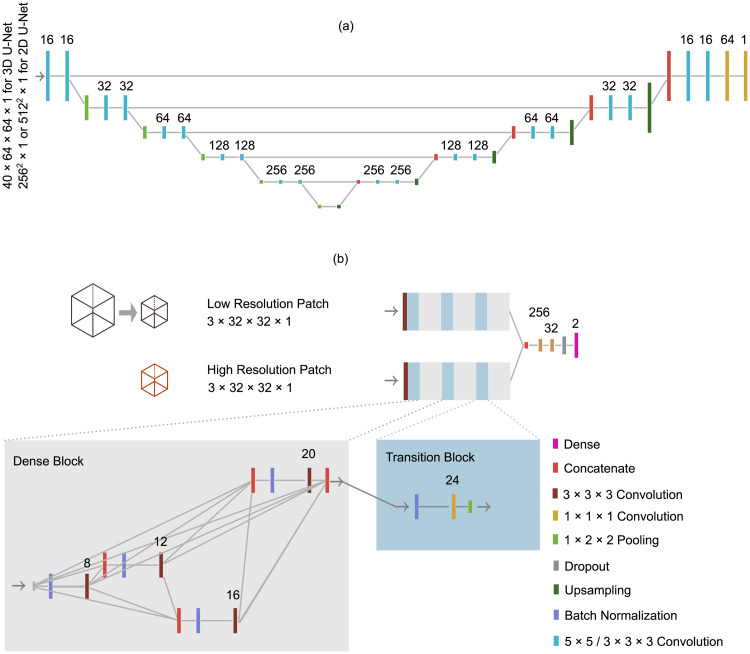
Network architectures. The proposed cranial cavity segmentation and region proposal architecture is displayed in (a). Hereby, for region proposal and 2D cranial cavity segmentation networks 5^2^ sized kernels are used. 3^3^ sized kernels are used for 3D U-Net based cranial cavity segmentation. The final dense layer uses a sigmoid activation function. The region classification architecture is illustrated in (b) and employs a dual-pathway network, which inputs patches extracted from the lesion border. It consists of multiple dense and transition blocks. Convolution layers use 3^3^ kernels for dense blocks and 1^3^ kernels for transition blocks. The final dense layer of uses a softmax activation function. Dropout layer rate was set to 0.2. For both architectures, numbers on top of layers display the number of convolution filters for convolutional layers or number of neurons for dense layers. Non-dotted line connections between two layers indicate flow from left to right. If not otherwise stated, convolutional layers utilize a ReLu activation function.

For cranial cavity segmentation, a 3D U-Net like architecture [9, 25] with an unweighted binary-crossentropy loss function is used in a first experiment (Fig 3a) and a 2D U-Net architecture [9] is used in another experiment. Rectified linear unit (ReLu) activations were chosen similar to the original U-Net studies.

For the 3D U-Net, volumes are first downsampled (40 × 64 × 64 voxel). After inference, a segmentation is retrieved and results are upsampled to the original volume size. However, due to the upsampling, the border of the segmentation is blurry. In order to obtain clean boundaries between soft-tissue and bones, voxels in the original volume are kept on a 11 pixel stripe around the segmentation border if their HU value is below 50 HU. The optimal distance was determined according to supplementary material S1 Text.

The HU value of 50 ensures to keep ischemic stroke lesions, which have profile values below 32 HU in the volume [26]. We emphasize that thresholding is only applied on the segmentation mask edges and that the relevant brain structures are extracted by the CNN and not by thresholding.

The 3D U-Net is compared to a 2D U-Net segmenting the cranial cavity on slice level. We used 5 × 5 sized kernels and hyper-parameters similar to the U-Net utilized for region-proposals (Fig 3a). Slices are resampled to a 512 × 512 resolution for input and retrieved segmentations are upsampled to the original slice resolution.

While it is beneficial to restrict the CNN input to the cranial cavity and remove irrelevant objects like the patient table, it is of interest whether a pixel-wise segmentation brings further performance improvements for stroke classification. Thus, for consecutive CNN training, we performed one experiment with the slices cropped around the cranial cavity on the x-y plane and another experiment with additional pixel-wise cranial cavity segmentation. Hereby, both experiments make use of the proposed cranial cavity segmentation method.

### Region proposal generation

For this stage, the aim is to identify areas where cerebral infarction has most likely occurred. A high sensitivity is desirable, as false-positives are eliminated in a later stage. A 2D U-Net derived architecture [9] is used (Fig 3a). The accuracy substantially depends on the loss function. Due to strong class imbalance on voxel-level, experiments were performed using a Dice loss function [27, 28]:

$$\begin{array}{c} \text{DICE}=\frac{2\sum_{p}S\left(p\right)y\left(p\right)}{\sum_{p}\left(S{\left(p\right)}^{2}+y{\left(p\right)}^{2}\right)}, \end{array}$$ (1)

given the predicted segmentation *S*(*p*) and the ground-truth *y*(*p*) for a pixel *p*. The dice loss is calculated per volume and averaged on batch level in order to penalize small and large lesions equally within a batch. Connected components with a size smaller than 0.2cm^3^ are removed by post-processing. This value was chosen below the size of the smallest stroke lesion in the segmented training and validation set.

### Region and volume classification

The segmentation network of the second stage only returns a binary segmentation on voxel level. To eliminate false positives and to generate a ROC curve, it is desirable to obtain a probability of how likely a single, connected lesion within the segmentation is classified as a stroke lesion. Hence, a third network is trained, which inputs patches extracted from the lesion borders and outputs a class (stroke/no stroke). The probability of a lesion is determined by averaging the probabilities of patches on that lesion (Fig 1). This patch-averaging technique was recently utilized on the ImageNet dataset [29] and is called *bag-of-local-features*. We consider this approach as beneficial, because the stroke lesion sizes have a high variance. To determine the stroke probability on a subject level, the maximum probability of all lesions of a volume is taken.

It is crucial to select the training data carefully. While it would be possible to extract non-stroke patches from random positions in healthy volumes, this is an inefficient approach, as it includes also patches from areas, which were classified as non-stroke by the stage 2 segmentation network. A better approach is to extract non-stroke patches from positions in healthy volumes, where the region proposal network of stage 2 yields a stroke segmentation (false-positives). For the other class, stroke patches are extracted from the ground-truth lesion annotations of the radiologist. This strategy is called hard-negative mining and was previously suggested for medical image analysis [13, 23].

To extract patches for training from the segmentation, connected lesions are found using a connected component labeling algorithm. Next, the border of the lesion is obtained using a Sobel filter on the segmentation. From each lesion, 70 random positions on this border are picked. Afterwards, 3D patches in two different resolutions (3 × 32 × 32 voxels and 3 × 64 × 64 voxels downsampled to 3 × 32 × 32 voxels) are extracted from these positions in the corresponding original volume and given as input to a dual-pathway architecture [12, 30]. We extend the dual-pathway architecture to a densely connected architecture [18, 31], an approach that was already applied in MRI brain lesion segmentation [32]. Densely connected networks consist of dense convolutional blocks and transition blocks. While convolutional layers of standard CNNs only input data from the previous layer, dense convolutional blocks contain multiple convolutional layers, whereby each layer input data from multiple previous convolutional layers in a respective block. Layers within a block have a rising number of filters, defined by the growth rate. Transition blocks limit the number of filters and apply a max-pooling operation. A detailed description can be found in [31]. Our proposed architecture is illustrated in Fig 3b. A categorical-crossentropy loss is used, as the two classes were hot-encoded. As the distribution consists of more healthy than stroke patches, unhealthy patches were oversampled [33] to ensure that all labels are equiprobable. ReLu activations were chosen similar to [31, 32].

In the test-phase, the three trained models are executed sequentially: first, the cranial cavity is segmented (optional) and region proposals are generated. From the retrieved proposed regions, 70 patches in two different resolutions from each lesion’s borders are extracted. For each patch, a probability is retrieved from the region proposal network. This returns 70 probabilities per lesion. To obtain a score on lesion-level, the mean of the 70 probabilities is calculated. After having calculated a score for each lesion, the score on case-level can be calculated by taking the maximum score out of all lesion scores.

### Comparison with end-to-end architectures

Two architectures that directly input the 3D volume and output a prediction are tested. First, a 3D DenseNet architecture, very similar to the end-to-end architecture used for comparison by De Fauw et al., [18] is tested: It consists of 5 dense convolutional blocks, each followed by a transition layer. The first dense block uses a 3 × 3 × 1 filter size for all convolutional layers and the remaining blocks use a 3 × 3 × 1 filter size for the first, second, third and fifth layer and a 1 × 1 × 3 filter size for the forth layer. A regularization rate of 10^−5^ is applied on convolutional layers. The first transition layer uses a 1 × 1 × 2 kernel for pooling, the remaining transition layers use a 2 × 2 × 2 kernel. The last layer is a global average pooling layer, followed by a dense layer. For each block, the initial filter count is 12 with a growth rate of 4.

The second architecture is similar to Arbabshirani [19] and consists of 5 convolutional layers (256, 64, 96, 128 and 128 filters), each followed by a max-pooling layer (two times 2 × 2 × 4 and three times 2 × 2 × 2 pooling). All convolutional layers use a 3 × 3 × 3 filter size. After the second layer we added a batch normalization layer, which is different from the original paper’s local response normalization layer. Last, 2 dense layers with 1000 units each are used and afterwards one dense layer with 2 units outputs the classification. For both architectures, all layers use a ReLu activation function, except the final layer which outputs the class. Here, a softmax activation is used and a categorical cross-entropy loss function is utilized, as the classes are hot-encoded. Augmentation includes width, height and rotation transformations.

### Measuring generalization performance and dataset bias

For image classification dataset bias is a factor to be considered [34]. As our CT scanners are from different vendors, it has to be ensured that the network does not classify its samples by features originating from the CT scanner model or patient age. CT scanner models use different post-processing kernels for image reconstruction and the brain structure of older patients is different from younger patients. Consequently both of these factors contribute to different textured brain tissue. As CNN training on large image databases such as ImageNet is often biased towards texture [35], the possibility of dataset bias for our classification method is examined by stratified sampling: For the first stratified set, for each CT scanner model as many stroke cases as non-stroke cases were used for testing. For the second set, classes of each patient are equiprobable for an age decade. Stratified and unstratified test set distributions are shown in Table 1.

### Network training

Optimizer, network and number of epochs for all networks are listed in Table 2, whereby Adam refers to the optimizer [36]. For 2D and 3D cranial cavity segmentation networks, 33 subjects were used for training, 4 for validation and 5 for test, whereby model weights with the best validation loss during training were used for the final model. As the task does not require detailed anatomical knowledge, the manual cranial cavity annotation was performed by a computer scientist and verified by a radiologist. Data augmentation was performed using rotation, shift and zoom transformations.

**Table 2:** Network parameters.

	Learning Rate	Optimizer	Epochs
2D Cranial Cavity Seg.	10^−4^	Adam	30
3D Cranial Cavity Seg.	10^−4^	Adam	320
Region Proposal	10^−4^	Adam	30
Patch Classification	10^−5^	Adam	5
E2E Hemorrhage	10^−5^	Adam	1000
E2E DenseNet	10^−5^	Adam	1000

Network training parameters for all utilized network architectures.

Total annotated data for training the region proposal network included 78 annotated CT scans with cerebral infarction. Training data included 62 scans, test data 8 and validation data 8 scans. Slices were resized to 256 × 256 pixels and during training the region proposal network, only slices with present stroke lesions were used. It was ensured that slices of a single volume only appear either in test, train or validation set.

A sigmoid activation function was used in the final layer. Final weights were obtained from the epoch with the best validation accuracy. For training the patch-classification network, patches of the minority class were oversampled.

Training data included patches from 53 segmented stroke cases and 133 healthy cases. Validation data contained patches from 26 stroke cases and 66 healthy cases. Final subject level test data included 186 samples in the unstratified and age-stratified test set and 150 samples in the scanner-stratified test set with equiprobable classes. Again, final weights were obtained from the epoch with the best validation accuracy. Batch normalization layers were initialized with a momentum of 0.99 and an epsilon parameter of 10^−3^ for all architectures. Weights were initialized using the glorot uniform initializer and biases were initialized with zeros. Models were implemented using Tensorflow [37] and Keras [38] and training was performed on a Titan Xp GPU.

### Statistical analysis

Results were analysed using a receiver operating characteristic (ROC) curve. Here, the true-positive rate is plotted against the false-positive rate for various discrimination thresholds. If the discrimination threshold decreases, the number of true-positives and false-positives increases. We calculated 95% confidence intervals (CI) of ROC curves using a bootstrap approach: The following experiment was repeated 1000 times: After selecting 100 random samples from the test set and calculating the AUC value of these samples in each experiment, we sorted the resulting AUCs of all experiments incrementally. Hereafter, we used the minimum and maximum value of the interval between 2.5% and 97.5% to obtain the 95% CI intervals.

## Results

Results were evaluated for cranial cavity segmentation, region proposals and subject-level classification, whereby subject-level classification results are of most interest. If not otherwise stated, numbers are provided in the format mean±standard deviation.

### Cranial cavity segmentation

Accuracy and time was evaluated for 3D and 2D U-Net based segmentations. Processing times for 2D and 3D U-Net were 1.2±0.05 and 2.99±0.11 seconds respectively. Corresponding dice scores were 0.9826±0.0033 for the 3D version and 0.9854±0.0033 for the 2D version. CT scan resolution for evaluation with respect to time was 34 × 592 × 592 voxels.

### Region proposals

**Fig 4:**
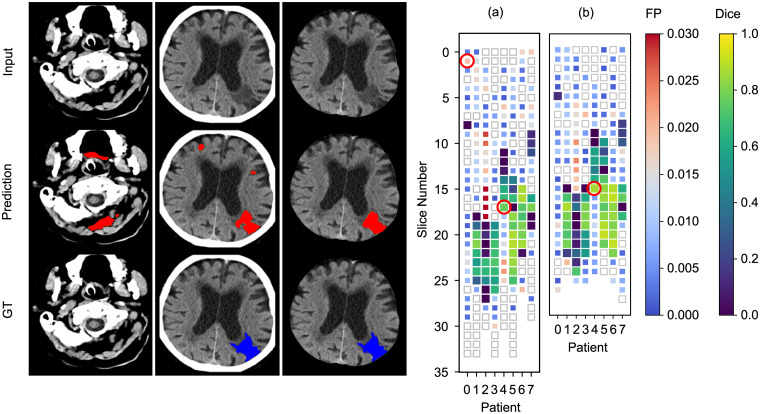
Region proposals evaluated on slice level using a Dice metric. The top row shows the original image, the second row the prediction and the third row the ground-truth. From left to right, the first image shows a segmentation with some false positive predicted lesions outside the brain for disabled cranial cavity segmentation. The second and third image shows a segmentation with mostly true positive predicted voxels whereby pixel-wise cranial cavity segmentation was disabled and enabled respectively. In the plots for disabled pixel-wise cranial cavity segmentation (a) and enabled pixel-wise cranial cavity segmentation (b), big squares indicate a stroke lesion in the ground-truth. Example slices are marked with a red circle in the plot. The square color shows the Dice score calculated from the ground-truth compared to the network’s prediction. Small squares with color show the ratio of false positive predictions on a slice (1.0 would indicate false positive voxels only). White squares with a silver border indicate only few or none false-positives, with coverage below 0.1 percent of the slice. The number of false-positive lesions is later reduced by the stage 3 classification network.

For stroke region proposals, the Dice loss was evaluated for enabled and disabled pixel-wise cranial cavity segmentation on slice level and illustrated in Fig 4. The latter experiment yielded a lower average dice score per volume (0.36±0.25 vs. 0.41±0.27) mostly due to more false positive detections in tissue distant from the brain.

### Subject level classification

**Fig 5:**
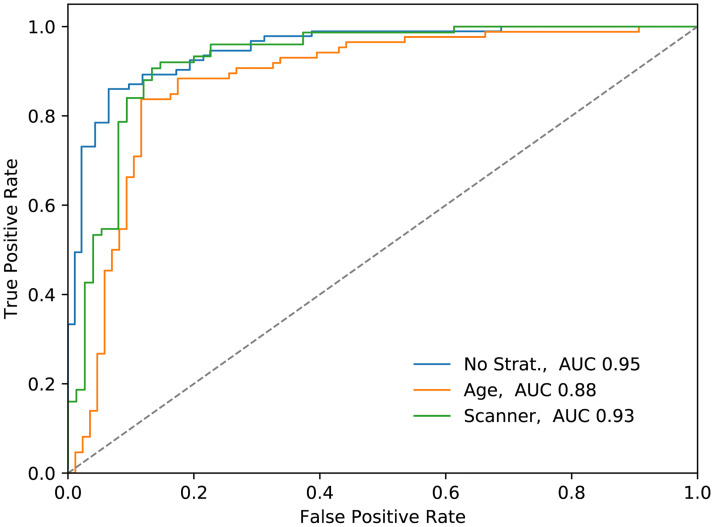
ROC curves for evaluation on the test set. Pixel-wise cranial cavity segmentation was disabled. Best results were retrieved for the unstratified test-set. Additional stratification to examine dataset bias was performed for age decade and CT scanner.

**Fig 6:**
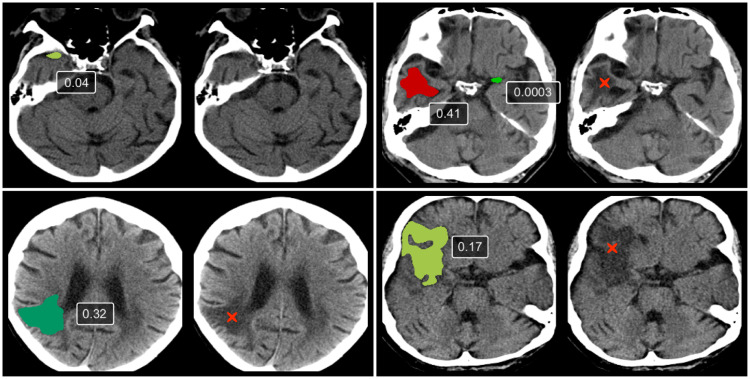
Scores of lesions predicted by the region classification network. Low scores (0.04 and 0.0003) are examples of false-positive lesions. These lesions received a low score in stage 3 after the stage 2 network had segmented the lesions. The remaining lesions are true positives. Lesion ground truth was verified by a radiologist (marked with “x”).

Final classification ROC curves for subject-level classification are shown in Fig 5. Visual output with lesion probabilities is shown in Fig 6.

Our method achieved an area under a curve (AUC) value of 0.95 for the unstratified test set, 0.88 for the age- and 0.93 for the scanner stratified test set without pixel-wise cranial cavity segmentation enabled. This indicates the algorithm is a little prone to dataset bias. Enabled pixel-wise cranial cavity segmentation delivered slightly worse results (0.94 for the unstratified test-set). By setting a threshold above which score lesions are considered as stroke, a confusion matrix can be generated. A confusion matrix for a threshold of 0.3 is shown in Table 3.

**Table 3:** Confusion matrix.

	True diagnosis		Total
	Positive	Negative	
Predicted Positive	73	4	77
Predicted Negative	20	89	109
Total	93	93	186

Confusion matrix with absolute numbers. Results were obtained for a ROC threshold of 0.3.

The performance of our architecture was compared to two end-to-end approaches previously utilized for hemorrhage detection and OCT classification (Table 4).

**Table 4:** ROC AUC results.

Experiment	AUC
No Strat.	0.95 (0.91–0.98)
Age Strat.,	0.88 (0.80–0.95)
Scanner Strat.	0.93 (0.86–0.97)
No Strat., C.S.	0.94 (0.89–0.98)
Age Strat., C.S.	0.89 (0.82–0.95)
Scanner Strat., C.S.	0.92 (0.86–0.97)
E2E Hemorrhage	0.80 (0.70–0.89)
E2E DenseNet	0.80 (0.71–0.88)

AUC values for subject-level classification of the unstratified test set from our approach compared to existing approaches. Confidence intervals are provided in brackets. Best results were retrieved using the unstratified test set (No Strat.). C.S. indicates pixel-wise cranial cavity segmentation enabled.

## Discussion

In this work, we proposed a fully-automatic multi-stage architecture for classification of non-contrast CT scans for cerebral infarction. Beside we contributed a method for cranial cavity segmentation, investigated the effect of dataset bias and compared our method to other CNN architectures.

Our method for cranial cavity segmentation reaches a similar Dice score of the non-deep learning approach of Patel et al. [22] (0.98) on a smaller test set, while being, to the best of our knowledge, notably faster (13.78±0.24 seconds vs. 6 minutes) for the same resolution. However, we emphasize that they did not use a GPU for calculation, which is utilized by deep learning frameworks and that performance depends on the available hardware. While pixel-wise enabled cranial cavity segmentation did not enhance classification performance in our experiments, we consider cropping the images around the cranial cavity segmentation as useful. It strongly reduces the number of input voxels for the region proposal network as it removes non-human objects such as the CT scanner table from the images.

For the second stage network, a U-Net architecture for region proposal generation is utilized. The choice of the architecture for region proposals depends on what kind of annotation is available. In case of box annotations, which can be obtained from pixel-wise annotations, it is also possible to use architectures such as R-CNN and its derivatives [39–41]. Mask R-CNN was already utilized for hemorrhage detection in previous work [17].

For classification, we investigated the effect of dataset bias. In the stratified test set, we observe a small decrease in performance after age and scanner stratification. This indicates that these parameters have influence on the networks decision. Hereby, it is a point to discuss, whether an unbiased decision for the patient age is desirable. A radiologist may unintentionally also yield a diagnosis more likely in older than in younger patients. Furthermore, we compared our method to existing methods: Only recently, CNN models which directly input the complete volume data instead of patches have been proposed. We evaluated two of these in our experiments, but did not achieve a performance comparable to our multi-stage method for cerebral infarction classification. This is identical to the findings of Lee et al., [16] who also reported a reduced performance using an end-to-end approach for hemorrhage detection compared to their multi-stage approach.

We believe the worse performance is due to two reasons: First, the end-to-end approaches are usually trained with more volumes, e.g. Arbabshirani et al. [19] used 37074 studies in the training dataset. Second, we believe that the morphological characteristics of the disease play a role: In OCT scans, as investigated by De Fauw. et al. [18], diseases usually cover a larger part of the volume, which makes it easier to detect features in lower resolutions (e.g. after pooling layers). For hemorrhage, affected areas can be discriminated from brain tissue more easily, as the HU value of affected areas is usually higher than remaining brain tissue. Both characteristics may have a positive impact on the convergence of the end-to-end network during the training process.

While multi-stage approaches are used for volume segmentation [42] and classification [17, 18], the idea of reducing input data for the second network using a prior network is the same for both tasks. In our case, these offer two advantages compared to end-to-end training using a single network: First, a lower number of volumes for training is required. And second, it helps the clinician and the developers to understand the CNN’s decision by examining the segmentation and the additional score for each lesion. This advantage was also outlined in previous work utilizing prior segmentation for classification tasks [17, 18]. In the stage 3 network, we utilized hard-negative mining, as proposed by [13, 23]. While in initial experiments we extracted healthy patches from random positions in healthy volumes, we found it crucial to extract patches from positions where the second stage network yielded a stroke segmentation in the presented study.

However, a disadvantage of the multi-stage approach compared to end-to-end approaches is that three networks instead of one network need to be trained. This results in a more complex routine for data extraction, as, for example patches need to be extracted for the classification network first. One more potential disadvantage is the need of pixel-wise segmented training data. This data can not be extracted from the PACS, but needs to be manually annotated by an expert radiologist, which is both, a time and cost intensive process.

In our pipeline, no manual interaction, such as region-of-interest selection, is needed, which is essential for fully automatic evaluation. A promising use case is to warn radiologists for false-negatives after examinations. Hereby, structured reporting, an uprising technology, shows great potential for clinical use [43] and would further allow seamless integration into clinical work flow. As the indication of an examination is already selected in a machine readable format by the user, a sub-process for automatic evaluation can be started in the background and co-evaluate the CT volume.

Future work includes the acquisition of more data and further optimzation of the network architecture. To further improve performance, we plan to use ensemble networks, similar to Fauw et al. [18], in which multiple networks with a identical architecture are trained and the prediction outcomes are averaged. In addition, we plan to perform further research on how different network parameters (such as the patch size) affect the network performance.

In conclusion, we presented a method for classification of brain CT scans for cerebral infarction as our main contribution. Additionally, we evaluated our method on a broad range of CT scanners and investigated the effect of dataset bias.

## Supporting information

S1 Text 3D U-Net border distance optimization. This file describes how clean segmentation boundaries for cranial cavity segmentation were obtained from 3D U-Net predictions.
(PDF)

S1 Datapoints Classification datapoints. This file contains prediction and groundtruth datapoints to calculate the ROC curves of the classification networks. Furthermore per-volume Dice scores of intermediate results are included.
(ZIP)
