# Supplementary material for: Towards subject-level cerebral infarction classification of CT scans using convolutional networks
Source: PLoS One. 2020 Jul 15;15(7):e0235765. doi: 10.1371/journal.pone.0235765 (PMC7363075; doi:10.1371/journal.pone.0235765)
Supplement: S1 Text — This file describes how clean segmentation boundaries for cranial cavity segmentation were obtained from 3D U-Net predictions. (PDF) [file pone.0235765.s001.pdf]

Supplementary Material 1
3D U-Net Border Distance Optimization

As the 3D cranial cavity segmentation yields a low resolution output which is upscaled, voxels around the border may contain bone voxels:

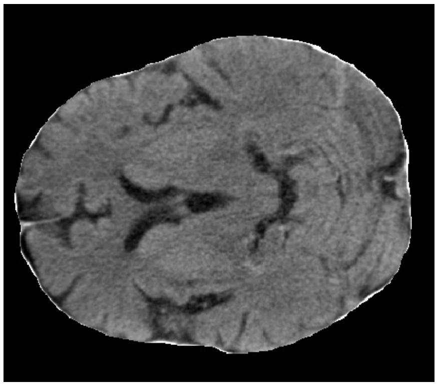

To avoid this, around the segmentation border voxels within a certain distance are kept if their HU value is below 50. To determine the optimal distance, we performed a parameter search. As such an optimization should never be done on the test-set, optimization was performed on the validation set. We tested the distances 3, 5, 7, 9, 11, 13, 15 pixels and disabled post-processing (Distance 0). We found 11 pixel to be the best distance, which yielded an average dice score of 0.9805 on the validation set. Consequently, this distance was used in the test set, where a Dice score of 0.9826 was achieved.

Detailed per-volume dice scores are shown in "3d_unet_distance_hyperparam.xlsx" tables in S2, which enlists Dice scores for all distances and validation set volumes.

Final results of the 3D cranial cavity segmentation were still worse than for the 2D method, so the 2D method, which does not require this post-processing step, should be favored.
